# Non-genomic Actions of Methylprednisolone Differentially Influence GABA and Glutamate Release From Isolated Nerve Terminals of the Rat Hippocampus

**DOI:** 10.3389/fnmol.2020.00146

**Published:** 2020-08-05

**Authors:** Rafael Neiva, Ana Caulino-Rocha, Fátima Ferreirinha, Maria Graça Lobo, Paulo Correia-de-Sá

**Affiliations:** ^1^Laboratório de Farmacologia e Neurobiologia – Departamento de Imuno-Fisiologia e Farmacologia, Instituto de Ciências Biomédicas de Abel Salazar (ICBAS), Universidade do Porto (UP), Porto, Portugal; ^2^Center for Drug Discovery and Innovative Medicines (MedInUP), Instituto de Ciências Biomédicas de Abel Salazar (ICBAS), Universidade do Porto (UP), Porto, Portugal

**Keywords:** methylprednisolone, glucocorticoid receptors, mineralocorticoid receptors, synchronous GABA and glutamate release, hippocampal nerve terminals

## Abstract

Corticosteroids exert a dual role in eukaryotic cells through their action via (1) intracellular receptors (slow genomic responses), or (2) membrane-bound receptors (fast non-genomic responses). Highly vulnerable regions of the brain, like the hippocampus, express high amounts of corticosteroid receptors, yet their actions on ionic currents and neurotransmitters release are still undefined. Here, we investigated the effect of methylprednisolone (MP) on GABA and glutamate (Glu) release from isolated nerve terminals of the rat hippocampus. MP favored both spontaneous and depolarization-evoked [^14^C]Glu release from rat hippocampal nerve terminals, without affecting [^3^H]GABA outflow. Facilitation of [^14^C]Glu release by MP is mediated by a Na^+^-dependent Ca^2+^-independent non-genomic mechanism relying on the activation of membrane-bound glucocorticoid (GR) and mineralocorticoid (MR) receptors sensitive to their antagonists mifepristone and spironolactone, respectively. The involvement of Na^+^-dependent high-affinity EAAT transport reversal was inferred by blockage of MP-induced [^14^C]Glu release by DL-TBOA. Depolarization-evoked [^3^H]GABA release in the presence of MP was partially attenuated by the selective P2X7 receptor antagonist A-438079, but this compound did not affect the release of [^14^C]Glu. Data indicate that MP differentially affects GABA and glutamate release from rat hippocampal nerve terminals via fast non-genomic mechanisms putatively involving the activation of membrane-bound corticosteroid receptors. Facilitation of Glu release strengthen previous assumptions that MP may act as a cognitive enhancer in rats, while crosstalk with ATP-sensitive P2X7 receptors may promote a therapeutically desirable GABAergic inhibitory control during paroxysmal epileptic crisis that might be particularly relevant when extracellular Ca^2+^ levels decrease below the threshold required for transmitter release.

## Highlights

- Methylprednisolone (MP) favors the spontaneous and depolarization-evoked release of Glu from rat hippocampal nerve terminals, without affecting GABA outflow;
- Facilitation of Glu release by MP is mediated by a Na^+^-dependent Ca^2+^-independent fast non-genomic mechanism altering high-affinity Glu transport through the plasma membrane;
- Depolarization-evoked GABA release in the presence of MP depends on activation of ATP-sensitive P2X7 receptors.

## Introduction

Glucocorticoids (GRs) are steroid hormones that are responsible for countless important regulatory functions in the human body (Joëls and Ronald de Kloet, 1994; Zhang et al., 2007; Groeneweg et al., 2012). Among the lifesaving corticosteroid drugs widely prescribed, methylprednisolone (MP) is used since 1955 and is listed as an Essential Medicine by the World Health Organization for the treatment of chronic inflammatory conditions, autoimmune diseases and allergic reactions, including several neurological disorders (Gumy et al., 2008; Kajiyama et al., 2010; Nicolaides et al., 2010). The usual daily dosing regimens indicated for MP vary significantly (from 15 to 20 mg to 120 mg) depending on the underlying disease condition. Higher dosing regimens of MP (e.g., 30 mg/kg IV, followed by repeated injections or continuous perfusions during 24 to 48 h) have been advocated in life-threatening edematous conditions, acute spinal cord injuries, and auto-immune rheumatic diseases. Although MP safety margin is high and side effects relatively rare (Bast et al., 2014; Tauheed et al., 2014), it may be detrimental for certain neurological conditions (reviewed in Parissis et al., 2017).

The hippocampus is a major structure of the human brain, which has been implicated in a variety of different functions, including declarative memory consolidation and formation of spatial memories. Like other limbic structures, the hippocampus is highly enriched in corticosteroid receptors, both GR and mineralocorticoid (MR) receptors (Joëls et al., 2012). This brain region is affected in several types of dementia and it is highly sensitive to pathological insults (e.g., hypoxia/ischemia, epileptic crisis, post-traumatic stress). Regarding the effects of GRs in the hippocampus, decreases in the number of interneurons in hippocampal CA3 and dentate gyrus have been observed after MP use for brain injury (Zhang et al., 2011). High doses of GRs are known to affect the induction of hippocampal long-term potentiation (LTP; Krugers et al., 2005; Maggio and Segal, 2007) and block synaptic strength and the formation of new synapses between CA3 and CA1 regions (Saito et al., 2016). Contrariwise, low to moderate doses of corticosterone may enhance LTP (Diamond et al., 1992). Recently, our group proposed MP (5–30 mg/kg, i.p., for 10 days) as a cognitive enhancer *in vivo* because it favored aversive memory persistence in adult rats, without any effect on exploring behavior, locomotor activity, anxiety levels and pain perception (de Vargas et al., 2017).

Despite the high number of studies in the literature aiming at exploring the effects of corticosteroids in the brain, their role is still controversial (Bartholome et al., 2004; Song and Buttgereit, 2006; Zhang et al., 2007; Rickard and Young, 2009; Groeneweg et al., 2012; Oliveira et al., 2015; Russo et al., 2016). The reason for this disparity may be attributed to insufficient knowledge about the exact molecular mechanism(s) of action of corticosteroids in the nervous tissue. Corticosteroids exert a dual role in eukaryotic cells acting via activation of (i) cytosolic receptors mediating slow genomic responses, and/or (ii) plasma membrane-bound receptors, resulting in fast changes in neuronal signaling, ion channel conductance and neurotransmitters release (non-genomic mechanisms). In a recent publication, we demonstrated that amplification of the neuromuscular transmission by MP (300 μM) involves activation of pre-synaptic facilitatory A_2*A*_ receptors by endogenous adenosine generated from ATP released by motor nerve terminals under resting conditions, leading to subsequent synaptic vesicles redistribution that favors acetylcholine exocytosis during high-frequency neuronal activation (Oliveira et al., 2015). In the hippocampus, data suggest that corticosteroids rapidly increase the mobility of post-synaptic membrane-bound glutamate receptors and facilitate the glutamate release probability from pre-synaptic neurons, thus strengthening the glutamatergic neurotransmission (Karst et al., 2005; Groc et al., 2008; Olijslagers et al., 2008; Wang and Wang, 2009; Zheng, 2009; Groeneweg et al., 2012). These findings are, however, far from being consensual, along with the fact that experimental studies addressing the effects of corticosteroids on inhibitory GABAergic neurotransmission are surprisingly lacking in literature.

Thus, this study was designed to investigate in parallel and under the same experimental conditions the mechanisms underlying the effect MP on resting and depolarization-evoked [^3^H]GABA and [^14^C]Glu release from isolated nerve terminals of the hippocampus of adult rats.

## Experimental Procedures

### Drugs and Solutions

2′(3′)-*O*-(4-Benzoylbenzoyl)adenosine 5′-triphosphate triethylammonium salt (BzATP), dexamethasone, GABA (γ-aminobutyric acid), mifepristone, spironolactone, NMDG (*N*-methyl-D-glucamine), EGTA (ethylene glycol-bis(2-aminoethylether)-*N*,*N*,*N*′,*N*′-tetraacetic acid) and aminooxyacetic acid were obtained from Sigma-Aldrich (St. Louis, MO, United States). L-Glutamic acid, A-438079 (3-[[5-(2,3-dichlorophenyl)-1*H*-tetrazol-1-yl] methyl]pyridine hydrochloride), DL-TBOA (DL-*threo*-β-benzyloxyaspartic acid), and VT were obtained from Tocris Bioscience (Bristol, United Kingdom). 1-(4,4-Diphenyl-3-butenyl)-3-piperidinecarboxylic acid hydrochloride (SKF-89976A) was obtained from Abcam (Cambridge, United Kingdom). MP was obtained from Pfizer (Puurs, Belgium). [^14^C]Glu and [^3^H]GABA were from American Radiolabeled Chemicals, Inc. (St. Louis, MO, United States). Stock solutions were stored as frozen aliquots. Dilutions of stock solutions were made daily and appropriate solvent controls were done. No statistically significant differences were observed in control experiments made with solvents at maximal concentrations compared to the physiological buffer.

### Animals

Hippocampi were obtained from Wistar Han rats of both genders (2–5 months old, Charles River^TM^, Barcelona, Spain). Animal care and experimental procedures were performed in consonance with the United Kingdom Animals (Scientific Procedures) Act 1986 and followed the European Communities Council Directive of 24 November 1986 (86/609/EEC) and the National Institutes of Health Guide for Care and Use of Laboratory animals (NIH Publications No. 80-23) revised 1996. All studies involving animals are reported in accordance with the ARRIVE guidelines for reporting experiments involving animals. The study was approved by the Ethics Committee and the Animal Welfare Responsible Organism of ICBAS-UP (Decision no 224/2017). Wistar rats were kept at a constant temperature (21°C) and a regular light (06.30–19.30 h)–dark (19.30–06.30 h) cycle, with food and water *ad libitum*. All efforts were made to minimize animal suffering and to reduce the number of animals used.

### Isolation of Nerve Terminals From the Rat Hippocampus

Nerve terminals were isolated as previously described (Helme-Guizon et al., 1998; modified in Bancila et al., 2009; Barros-Barbosa et al., 2015). Briefly, brain hemispheres were separated and hippocampi were dissected out and gently homogenized in cold oxygenated (95% O_2_ and 5% CO_2_) Krebs solution (in mM: glucose 5.5, NaCl 136, KCl 3, MgCl_2_ 1.2, Na_2_HPO_4_ 1.2, NaHCO_3_ 16.2, CaCl_2_ 0.5, pH 7.40). Homogenates were filtered through a nylon filter (mesh size 100 μm). The filtrate was left to sit during 30–45 min until formation of a pellet, which was re-suspended into Krebs solution and left at room temperature. Protein concentration determined by the bicinchoninic acid method (Pierce^TM^ bicinchoninic acid protein assay, Thermo Scientific, Rockford, IL, United States) was adjusted to 6.25 mg protein mL^–1^.

Re-sealed nerve terminal membranes isolated from the rat hippocampus using this methodology allows processing larger amounts of tissue compared to synaptosomal preparations, while taking up and release [^3^H]GABA and [^14^C]glutamate via Na^+^-dependent high-affinity transporters with a similar kinetics. This method is relevant in order to take full advantage of scarce human brain tissue (from cadaveric samples and surgical resections of epileptic foci) often tested in parallel by our group (see Barros-Barbosa et al., 2015, 2016, 2018).

### [^3^H]GABA and [^14^C]Glu Release Experiments

Isolated nerve terminals were incubated with [^3^H]GABA (0.25 μCi mL^–1^; 70 Ci mmol^–1^; 0.5 μM) and [^14^C]Glu (0.25 μCi mL^–1^; 0.270 Ci mmol^–1^; 10 μM), for 10 min at 37°C, and their release was measured simultaneously. Aliquots of nerve terminals suspension were layered onto glass fiber filters (Merck Millipore, Cork, Ireland), which were mounted in 365 μL chambers of a semi-automated 12-sample superfusion system (SF-12 Suprafusion 1000, Brandel, Gaithersburg, MD, United States). The filters containing the sample were superfused at a flow rate of 0.5 mL min^–1^, at 37°C, with a physiological solution containing (in mM): NaCl 128, MgCl_2_ 1.2, KCl 3, glucose 10, HEPES–Na 10 (pH = 7.4), CaCl_2_ 2.2, and aminooxyacetic acid 0.01, an inhibitor of tissue GABA breakdown by 4-aminobutyrate aminotransferase (GABA-T). After a 26 min equilibration period, 2-min fractions were automatically collected, using the SF-12 suprafusion system. Eight (S1) and twenty-six (S2) min after starting samples collection, isolated nerve terminals were depolarized with a solution containing high KCl (15 mM) or VT (5 μM, a Na^+^ channel activator) during 2 min; this was done by changing the inlet tube from one flask to another containing the depolarizer agent. High KCl and VT are the most common strategies to depolarize the plasma membrane of isolated nerve terminals as they allow to differentiate between pre-synaptic Na^+^ channel-mediated responses (Adam-Vizi and Ligeti, 1986; Costa et al., 2006). VT acts as a neurotoxin through binding to site 2 of voltage-gated sodium channels resulting in their persistent activation.

Methylprednisolone was added 15 min before S2. Solutions without Ca^2+^ or Na^+^ and containing drug modifiers (e.g., transport inhibitors, receptor antagonists) were applied from the beginning of samples collection, so that they were present throughout the testing period including S1 and S2; under such conditions, the normalized [^3^H]GABA and [^14^C]Glu release in S2 compared to S1 was not different (*P* > 0.05) from that obtained in control conditions (with no drugs added). The radioactive content of collected fractions and that remaining in the filters at the end of the protocol was measured by liquid scintillation spectrometry (TriCarb2900TR, Perkin Elmer, Boston, MA, United States).

### SDS–PAGE and Western Blot Analysis

Total membrane lysates and nerve terminals isolated from the rat hippocampus were homogenized in Radio Immuno Precipitation Assay (RIPA) buffer containing: Tris–HCl (pH 7.6) 25 mM, NaCl 150 mM, sodium deoxycholate 1%, triton-X-100 1%, SDS 0.1%, EDTA 5 mM and a protease inhibitor cocktail (Sigma-Aldrich, St. Louis, MO, United States). The protein content of the samples was evaluated using the BCA method. Samples were solubilized at 70°C in SDS reducing buffer [Tris–HCl (pH 6.8) 125 mM, SDS 4%, bromophenol blue 0.005%, glycerol 20%, and 2-mercaptoethanol 5%] for 10 min, subjected to electrophoresis in 12.5% SDS–polyacrylamide gels and electrotransferred onto PVDF membranes (Merck MilliPore, Temecula, CA, United States). Membranes were blocked for 1 h in Tris-buffered saline [TBS; in mM: Tris–HCl 10 (pH 7.6), NaCl 150] containing Tween 20 0.05% and BSA 5% and, subsequently, incubated overnight, at 4°C, with primary antibodies: mouse anti-synaptophysin (1:1000, Chemicon, Temecula, CA, United States) and mouse anti-GFAP (1:500, Chemicon, Temecula, CA, United States). Membranes were washed three times for 10 min in 0.05% Tween 20 in TBS and then incubated with horseradish anti-rabbit or anti-mouse peroxidase-conjugated secondary antibodies for 120 min, at room temperature. The antigen–antibody complexes were visualized by chemiluminescence with the Immun-Star WesternC Kit (Bio-Rad Laboratories, Hercules, CA, United States) using the ChemiDoc MP imaging system (Bio-Rad Laboratories, Hercules, CA, United States). Gel band image densities were quantified with ImageJ (National Institute of Health, United States).

### Immunofluorescence Confocal Microscopy

Immunofluorescent staining and confocal microscopy analysis was performed as previously described (Barros-Barbosa et al., 2015, 2016). Brain samples were fixed in 4% paraformaldehyde in phosphate-buffered saline (PBS; in mM: NaCl 137, KCl 2.6, Na_2_HPO_4_ 4.3, KH_2_PO_4_ 1.5; pH = 7.4) for about 48 h (4°C), cryopreserved in 30% sucrose in PBS and stored in a tissue freezing medium at −80°C. Free-floating 30-μm brain slices were incubated for 1 h, at room temperature, with blocking buffer I (fetal bovine serum 10%, BSA 1%, triton X-100 0.5%, NaN_3_ 0.05%) and subsequently incubated overnight, at 4°C, with the following primary antibodies: rabbit anti-P2X7 receptor (1:50, #APR004, Alomone, Jerusalem, Israel), mouse anti-GFAP (1:350, Chemicon, Temecula, CA, United States), goat anti-VAMP-1 (1:20, R&D Systems, Minneapolis, MN, United States) diluted in blocking buffer II (fetal bovine serum 5%, BSA 0.5%, triton X-100 0.5%, NaN_3_ 0.05% in PBS). Sections were rinsed in PBS supplemented with triton X-100 0.5% (three cycles of 10 min) and incubated for 120 min with species-specific secondary antibodies conjugated with fluorescent dyes (donkey anti-rabbit IgG Alexa Fluor 488, donkey anti-mouse IgG Alexa Fluor 568; donkey anti-goat Alexa 633) diluted in blocking buffer II, at room temperature. After rinsing in PBS, slices were mounted on optical-quality glass slides using VectaShield (Vector Labs, Peterborough, United Kingdom) as mounting media. Observations were performed with a laser scanning confocal microscopy (Olympus FV1000, Tokyo, Japan). Controls were performed by following the same procedure but replacing the primary antibodies with the same volume of blocking buffer II. Images were analyzed using the Olympus Fluoview 4.2 Software (Olympus FV1000, Tokyo, Japan). Co-localization was assessed by calculating the staining overlap and the Pearson’s coefficient (ρ) for each confocal micrograph stained with two fluorescent dyes. Overlap between two stainings gives a value between +1 and 0 inclusive, where 1 is total overlap and 0 is no overlap. ρ is a measure of the linear correlation between two variables (stainings), giving a value between +1 and −1 inclusive, where 1 is total positive correlation, 0 is no correlation, and −1 is total negative correlation.

### Data Presentation and Statistical Analysis

[^14^C]Glu and [^3^H]GABA release by isolated nerve terminals was obtained as CPMs in function of time (min) obtained by the digital conversion of the intensity of radiation emitted by β particles detected by the liquid scintillation analyzer in channels 1 and 2, which correspond to the specific range of β energies (counting windows) for [^14^C] and [^3^H], respectively. Correction for efficiency and background are needed to convert CPMs into DPMs, the number of decay events that actually occurred; this was done using the expressions 1 and 2 presented below.

(1) $$C=\frac{N_{1}-N_{2}\,\left(h_{1}/h_{2}\right)}{c_{1}-c_{2}\,\left(h_{1}/h_{2}\right)}$$

(2) $$H=\frac{N_{2}-N_{1}\,\left(c_{2}/c_{1}\right)}{h_{2}-h_{1}\,\left(c_{2}/c_{1}\right)}$$

*C* = *carbon DPMs in sample*

*H* = *tritium DPMs in sample*

*c_1_* = *carbon-14 efficiency in channel 1*

*c_2_* = *carbon-14 efficiency in channel 2*

*h_1_* = *tritium efficiency in channel 1*

*h_2_* = *tritium efficiency in channel 2*

*N_1_* = *CPMs in channel 1*

*N_2_* = *CPMs in channel 2*

The area of the peak corresponding to the release of [^3^H]GABA and [^14^C]Glu was calculated as the sum of the differences between the total radioactivity present in 2–3 samples collected after stimulus (e.g., KCl, VT) application and the basal leakage of the corresponding isotope. Baseline values during stimulus influence were inferred by linear regression of the radioactivity decay immediately before stimulus and after its return to baseline. Thus, the action of test drugs on evoked [^3^H]GABA and [^14^C]Glu release during S2 was normalized by the effect of the depolarizing conditions alone obtained in S1 of the same experiment (S2/S1 ratios).

The results are expressed as mean ± SEM, with *n* (showed in graphs) indicating the number of animals. Statistical analysis of data was carried out using GraphPad Prism 7.05 software (La Jolla, CA, United States). One-way ANOVA (uncorrected Fisher’s Least Significant Difference or Tukey’s multiple comparison test, with single pooled variance) or two-way ANOVA (followed by the Dunnett’s multicomparison test) were used when appropriate. Unpaired Student’s *t*-test was used to compare the ratios between GFAP and synaptophysin protein densities (immunoblots) in total cell lysates and isolated nerve terminals obtained from the same hippocampus in three different animals. *P* < 0.05 (two tailed) values were considered to show significant differences between means.

## Results

### Nerve Terminals Isolated From Rat Hippocampal Homogenates Are Highly Enriched in the Presynaptic Marker, Synaptophysin, With No Contamination by Glial Subcellular Particles

**FIGURE 1:**
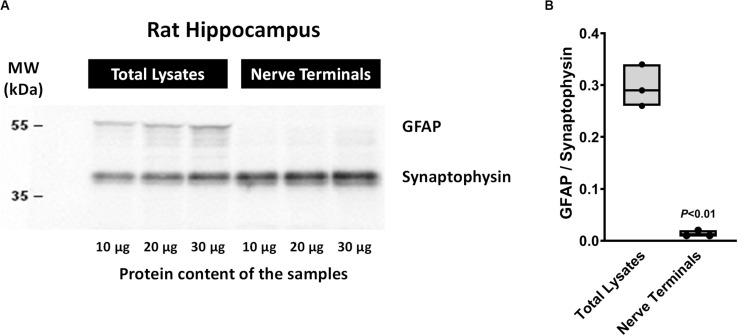
Western blot analysis of synaptophysin and glial fibrillary acidic protein (GFAP) contents in total cell lysates and isolated nerve terminals of the rat hippocampus by the method of Helme-Guizon et al. (1998; method modified in Bancila et al., 2009). Panel **(A)** shows representative immunoblots of three distinct animals loaded with different protein amounts (10, 20, and 30 μg). In panel **(B)** shown are floating bars (min to max, line at median) representing the ratios between GFAP and synaptophysin protein densities obtained in total cell lysates and isolated nerve terminals of three distinct animals. *P* < 0.05 (unpaired Student’s *t*-test) represents a significant difference compared to total cell lysates. Please note that under the present experimental conditions synaptophysin-immunoreactivity indicates that rat hippocampal cell fractions are highly enriched in nerve terminals, whereas the lack of GFAP immunoreactivity denotes very little (if at all) contamination by glial subcellular particles (gliosomes).

The method used for nerve terminals isolation described by Helme-Guizon et al. (1998; modified in Bancila et al., 2009; Barros-Barbosa et al., 2015) yields greater amounts of nerve terminals from rat hippocampal homogenates with very little (if at all) contamination by glial subcellular particles (gliosomes). This was demonstrated by the high density of synaptophysin (a typical nerve terminal marker) compared to the astrocytic glial fibrillary acidic protein (GFAP) content detected in the nerve terminals fraction *vis a vis* that found in total hippocampal cell lysates by Western blot analysis (Figure 1).

### Methylprednisolone Increases the Resting and Depolarization-Evoked Release of Glu From Hippocampal Nerve Terminals via Fast Non-genomic Mechanisms, Without Affecting the GABA Outflow

**FIGURE 2:**
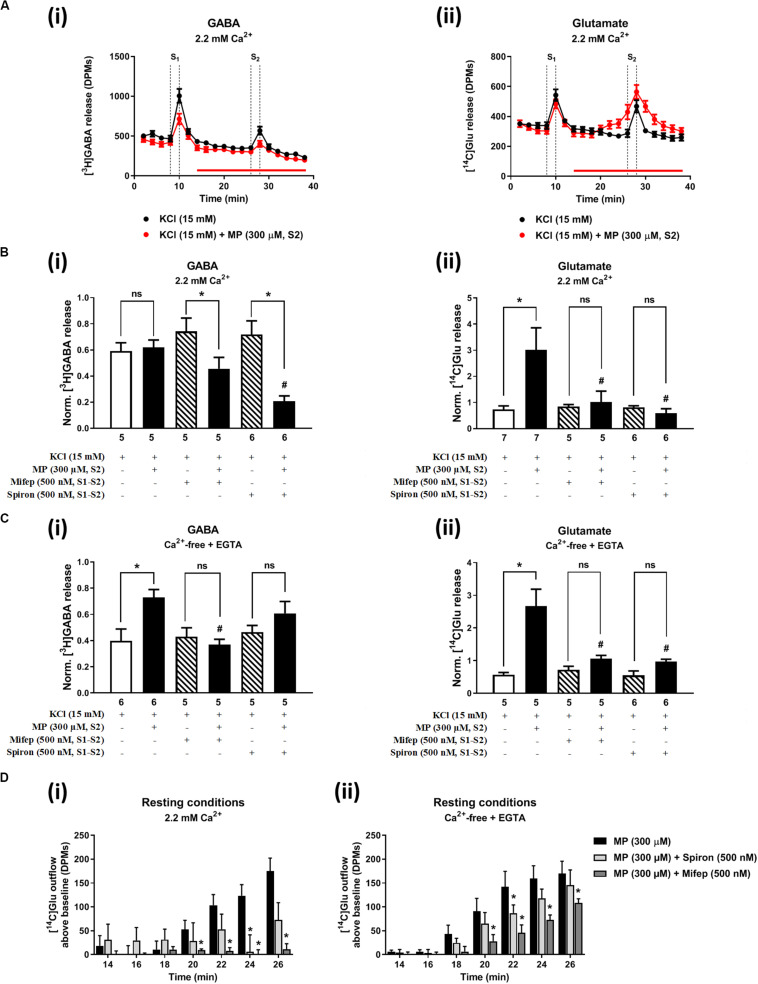
Methylprednisolone (MP) increases the resting and high KCl-evoked release of [^14^C]Glu from isolated nerve terminals of the rat hippocampus, without affecting the [^3^H]GABA outflow. Panel **(A)** shows [^3^H]GABA **(Ai)** and [^14^C]glutamate **(Aii)** outflow from rat hippocampal nerve terminals over time; nerve terminals were depolarized twice (S1 and S2) with high KCl (15 mM) applied for 2 min (dotted vertical lines). In panels **(B,C)**, ordinates represent the normalized [^3^H]GABA **(Bi,Ci)** and [^14^C]Glu **(Bii,Cii)** release (S2/S1 ratios) from hippocampal nerve terminals depolarized with high KCl (15 mM) in normal Ca^2+^ (2.2 mM, **B**) and in Ca^2+^- free (plus EGTA, 1 mM, **C**) conditions. MP (300 μM) was applied to the superfusion fluid 15 min before S2 (red horizontal line) either in the absence or in the presence of mifepristone (Mifep, 500 nM) and spironolactone (Spiron, 500 nM); corticosteroid receptor antagonists were present throughout the assay, including S1 and S2. Panel **(D)** illustrates the outflow of [^14^C]Glu above baseline over time caused by MP (300 μM) in the absence and in the presence of mifepristone (Mifep, 500 nM) or spironolactone (Spiron, 500 nM) in normal Ca^2+^ (2.2 mM, **Di**) and in Ca^2+^- free (plus EGTA, 1 mM, **Dii**) conditions. The results are expressed as mean ± SEM; the *n*, number of individual experiments per condition is shown below each bar. In panels **(B,C)**, *P < 0.05 and ^#^*P* < 0.05 (one-way ANOVA, uncorrected Fisher’s LSD with single pooled variance) represent significant differences compared to control conditions (i.e., drugs in S1 and S2) and to the effect of MP alone, respectively; ns, non-significant; the computed *F* ratios were 5.40 **(Bi)**, 4.05 **(Bii)**, 13.19 **(Ci)** and 6.59 **(Cii)**. In panel **(D)**, *P < 0.05 (two-way ANOVA, followed by the Dunnett’s multicomparison test) represent significant differences as compared to the effect of MP alone; the computed F ratios for rows (baseline variation) and columns (test groups) were 2.72 and 11.72 for **(Di)** and 26.24 and 12.66 for **(Dii)**, respectively.

**FIGURE 3:**
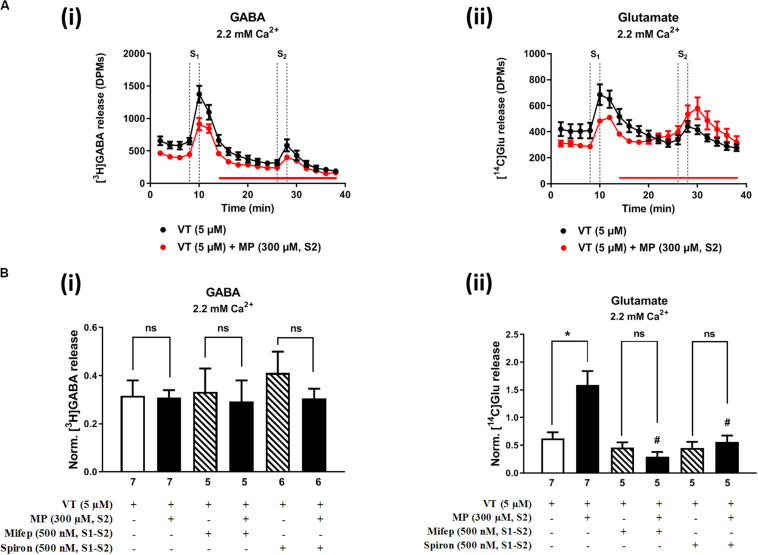
Methylprednisolone (MP) increases VT-evoked release of [^14^C]Glu from isolated nerve terminals of the rat hippocampus, without affecting the [^3^H]GABA outflow. Panel **(A)** shows [^3^H]GABA **(Ai)** and [^14^C]glutamate **(Aii)** outflow from rat hippocampal nerve terminals over time; nerve terminals were depolarized twice (S1 and S2) with VT (5 μM) applied for 2 min (dotted vertical lines). In panel **(B)**, ordinates represent the normalized [^3^H]GABA **(Bi)** and [^14^C]Glu **(Bii)** release (S2/S1 ratio) from hippocampal nerve terminals depolarized with VT (5 μM). MP (300 μM) was applied to the superfusion fluid 15 min before S2 (red horizontal line) either in the absence or in the presence of mifepristone (Mifep, 500 nM) and spironolactone (Spiron, 500 nM); corticosteroid receptor antagonists were present throughout the assay, including S1 and S2. The results are expressed as mean ± SEM; the *n*, number of individual experiments per condition is shown below each bar. In panel **(B)**, *P < 0.05 and ^#^*P* < 0.05 (one-way ANOVA, uncorrected Fisher’s LSD with single pooled variance) represent significant differences compared to control conditions (i.e., drugs in S1 and S2) and to the effect of MP alone, respectively; ns, non-significant; the computed *F* ratios were 0.44 **(Bi)** and 10.51 **(Bii)**.

Figures 2A, 3A show the time course of synchronous [^3^H]GABA and [^14^C]Glu outflow from rat hippocampal nerve terminals depolarized with high KCl (15 mM) or VT (5 μM), respectively. The depolarizing agents were applied twice (S1 and S2) during 2 min either in the absence or in the presence of MP (300 μM), which contacted with rat hippocampal isolated nerve terminals for at least 15 min before S2. Immediately after application, MP (300 μM) progressively increased the basal release of [^14^C]Glu, without affecting [^3^H]GABA outflow (Figures 2A, 3A; see Figure 2D for more detail). MP (300 μM) could still further increase depolarization-evoked [^14^C]Glu release by about three-fold compared to control conditions, but the same was not observed regarding the release of [^3^H]GABA (Figures 2B, 3B). The facilitatory effect of MP on KCl-induced [^14^C]Glu release was concentration-dependent (10–300 μM; with effects ranging from 16 to 336%, *n* = 5–6) and mimicked by the long-acting corticosteroid, dexamethasone, applied at an approximate equivalent concentration (60 μM, *n* = 5), but it was independent of the depolarizing agent, either high KCl (Figure 2B) or VT (Figure 3B).

The hippocampus expresses high amounts of corticosteroid receptors (de Vargas et al., 2017), but so far no attempt has been made to discriminate the effect of GR (low affinity) and MR (high affinity) receptors on transmitters release in this brain region. Pre-treatment of hippocampal nerve terminals either with mifepristone (500 nM) or with spironolactone (500 nM) to block GR and MR, respectively, prevented the facilitatory effect of MP (300 μM) on evoked [14C]Glu release independently of the depolarizing agent being used, either high KCl (Figure 2Bii) or VT (Figure 3Bii). Mifepristone (500 nM) blocked more efficiently than spironolactone (500 nM) the enhancing effect of MP (300 μM) on the resting [14C]Glu outflow (Figure 2Di), thus suggesting that GRs may have a predominant effect over the MR in the resting release of [^14^C]Glu. Unexpectedly, MP (300 μM) decreased KCl-induced [^3^H]GABA release in the presence of mifepristone (500 nM), and even more when spironolactone (500 nM) was used (Figure 2Bi), but no such effect was observed when VT was used to depolarize hippocampal nerve terminals (Figure 3Bi).

It is worth noting that, on their own, mifepristone (500 nM) and spironolactone (500 nM) did not significantly (*P* > 0.05) affect [^3^H]GABA and [^14^C]Glu outflow from rat hippocampal nerve terminals, both during resting conditions and following plasma membrane depolarization with high KCl (Figure 2B) or VT (Figure 3B). The S2/S1 ratio of KCl-induced [^3^H]GABA release in the presence of mifepristone (500 nM) and spironolactone (500 nM) applied 15 min before S2 was 0.63 ± 0.14 (*n* = 6) and 0.58 ± 0.03 (*n* = 5) vs. 0.60 ± 0.06 in control conditions (*P* > 0.05); likewise, these drugs also did not significantly modify (*P* > 0.05) the S2/S1 ratio regarding the release of [^14^C]Glu measured in the same samples (0.85 ± 0.16, *n* = 6 and 0.73 ± 0.09, *n* = 5, respectively), compared to the control situation when no drugs were added to the incubation fluid (0.78 ± 0.13, *n* = 7).

### Influence of External Ca^2+^ on Methylprednisolone-Induced Changes in Glu and GABA Outflow From Hippocampal Nerve Terminals

Performing experiments in Ca^2+^-free solutions (plus the Ca^2+^ chelator, EGTA 0.1 mM) restrain vesicular transmitter exocytosis and aim at mimicking conditions of paroxysmal neuronal activity verified during and after physiological and pathological brain events (such as epileptic seizures) where extracellular Ca^2+^ may fall up to 90% of the initial levels (Engelborghs K. et al., 2000; Engelborghs S. et al., 2000).

**FIGURE 4:**
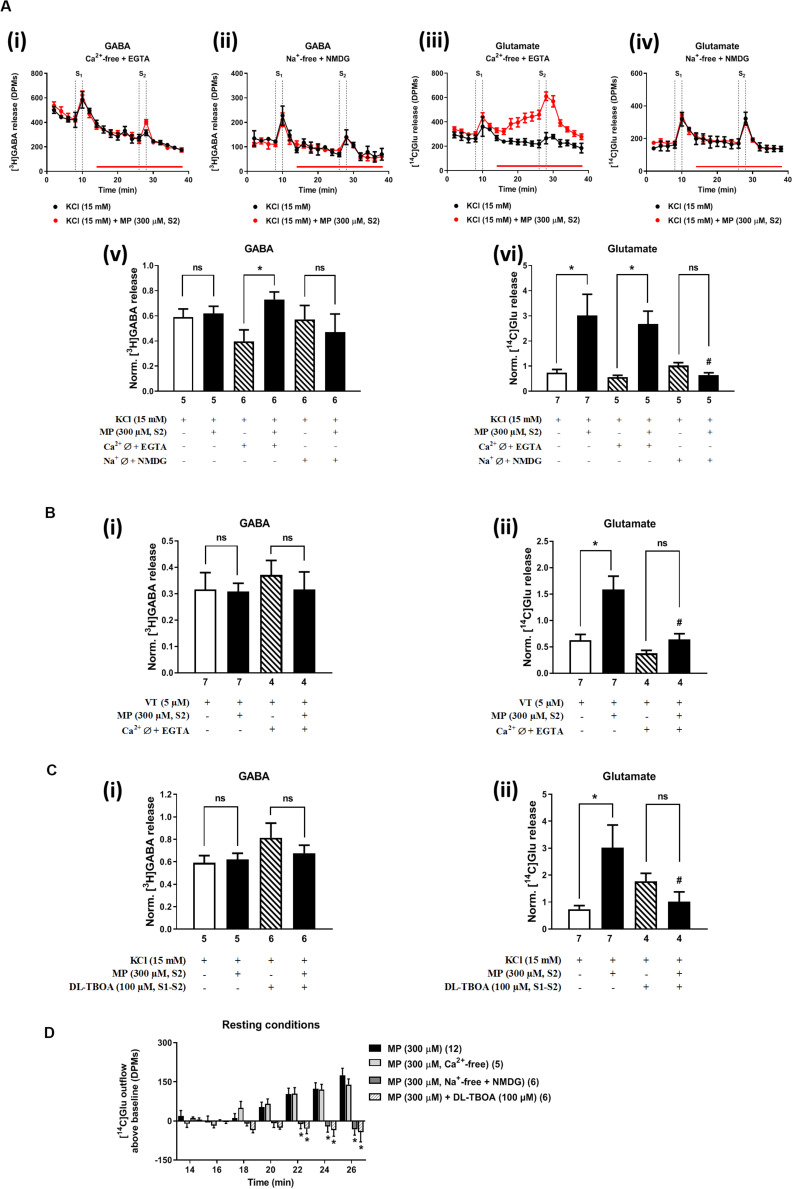
Influence of Ca^2+^ and Na^+^ withdrawal from the incubation fluid on the facilitatory effect of methylprednisolone (MP) on [^3^H]GABA and [^14^C]Glu release from resting and depolarized nerve terminals of the rat hippocampus. In panels **(A–C)**, ordinates represent the normalized [^3^H]GABA **(i)** and [^14^C]Glu **(ii)** release (S2/S1 ratio) from hippocampal nerve terminals depolarized with high KCl (15 mM, **A,C**) or VT (5 μM, **B**). MP (300 μM) was applied to the superfusion fluid 15 min before S2; solutions without Ca^2+^ (plus EGTA, 1 mM) or Na^+^ (which was replaced by NMDG, 128 mM) and containing DL-TBOA (100 μM) were applied from the beginning of sample collection, so that they were present throughout the assay, including S1 and S2. Panel **(D)** illustrates the outflow of [^14^C]Glu above baseline over time caused by MP (300 μM) in the absence and in the presence of superfusion solutions containing no added Ca^2+^ (plus EGTA, 1 mM), no added Na^+^ (replaced by NMDG, 128 mM) and DL-TBOA (100 μM). The results are expressed as mean ± SEM; the *n*, number of individual experiments per condition is shown below each bar. In panels **(A–C)**, *P < 0.05 and ^#^*P* < 0.05 (one-way ANOVA, uncorrected Fisher’s LSD with single pooled variance) represent significant differences compared to control conditions (i.e., Ca^2+^-/Na^+^-free conditions or DL-TBOA in S1 and S2) and to the effect of MP alone, respectively; ns, non-significant; the computed *F* ratios were 1.01 **(Av)**, 4.36 **(Avi)**, 0.36 **(Bi)**, 12.67 **(Bii)**, 1.22 **(Ci)** and 3.25 **(Cii)**. In panel **(D)**, *P < 0.05 (two-way ANOVA, followed by the Dunnett’s multicomparison test) represent significant differences as compared to the effect of MP in control conditions; the computed F ratios for rows (baseline variation) and columns (test groups) were 3.51 and 26.14, respectively.

In Ca^2+^-free media (plus EGTA 0.1 mM), MP (300 μM) could still progressively increase the release of [^14^C]Glu from resting hippocampal nerve terminals (Figures 4Aiii,D), without affecting the basal [^3^H]GABA outflow. Removal of Ca^2+^ from the incubation solution (plus EGTA 0.1 mM) also failed to affect MP (300 μM)-induced facilitation of [^14^C]Glu release evoked by high KCl (15 mM) depolarization (Figures 4Aiii,vi), but it significantly diminished the facilitatory effect of the corticosteroid when VT (5 μM) was used as depolarizing agent (Figure 4Bii). In Ca^2+^-free conditions (plus EGTA 0.1 mM), MP (300 μM) facilitated the release of [^3^H]GABA from hippocampal nerve terminals depolarized with high KCl (15 mM) [Figures 4Ai,v], but not when VT (5 μM) was used instead (Figure 4Bi).

The facilitatory effect of MP (300 μM) on high KCl-induced [^3^H]GABA and [^14^C]Glu release in Ca^2+^-free media (plus EGTA 0.1 mM) was abrogated by the GR antagonist, mifepristone (500 nM) (Figures 2Ci,ii, respectively), while pre-treatment with spironolactone (500 nM) only significantly (*P* < 0.05) decreased MP (300 μM)-induced facilitation of [^14^C]Glu release (Figure 2Cii) with a minor effect on [^3^H]GABA outflow (Figure 2Ci). Like that observed in normal Ca^2+^ conditions (Figure 2Di), mifepristone (500 nM) blocked more efficiently than spironolactone (500 nM) the basal [^14^C]Glu outflow promoted by MP (300 μM) in Ca^2+^-free media (Figure 2Dii).

### Dissipation of the Na^+^ Gradient Across the Plasma Membrane Prevents Facilitation of KCl-Evoked Glu Release by Methylprednisolone, Without Affecting the GABA Outflow

The transmembrane Na^+^ gradient is required to take up GABA and Glu from the extracellular milieu by high-affinity amino-acid transporters (GAT1 and EAATs). Substitution of Na^+^ with NMDG (128 mM) in the incubation medium transiently increased [^3^H]GABA and [^14^C]Glu outflow by rat cortical synaptosomes (see e.g., Wu et al., 2006). Previously, we showed that disruption of the transmembrane Na^+^ gradient leading to a reversal of amino-acid transporters to operate in the releasing mode can affect differentially the outflow of [^3^H]GABA and [^14^C]Glu (Barros-Barbosa et al., 2018).

Replacement of extracellular Na^+^ with NMDG (128 mM) failed to affect KCl-induced [^3^H]GABA release in the presence of MP (300 μM) (Figures 4Aii,v), but it fully prevented the facilitatory effect of the corticosteroid on [^14^C]Glu outflow, both under resting conditions (Figures 4Aiv,D) and during depolarization of hippocampal nerve terminals with high KCl (15 mM) (Figures 4Aiv,vi). Taking this into account, we questioned whether blockage of high-affinity EAAT transporters working in the reverse mode with DL-TBOA (100 μM) could also prevent MP (300 μM)-induced facilitation of [^14^C]Glu release from rat hippocampal nerve terminals. As a matter of fact, DL-TBOA (100 μM) significantly (*P* < 0.05) attenuated the facilitatory effect of MP (300 μM) on [^14^C]Glu release, both under resting conditions (Figure 4D) and during depolarization of rat hippocampal nerve terminals with high KCl (15 mM) (Figure 4Cii), a situation that mimicked the effect observed by replacing Na^+^ with NMDG (128 mM) in the incubation medium (Figure 4Avi). No changes in the coincidental release of [^3^H]GABA were observed under the latter experimental conditions (Figures 4Av,Ci).

### Blockade of the ATP-Sensitive P2X7 Receptor Decreases Depolarization-Evoked [^3^H]GABA Release From Hippocampal Nerve Terminals in the Presence of Methylprednisolone, Without Affecting the Release of [^14^C]Glu

**FIGURE 5:**
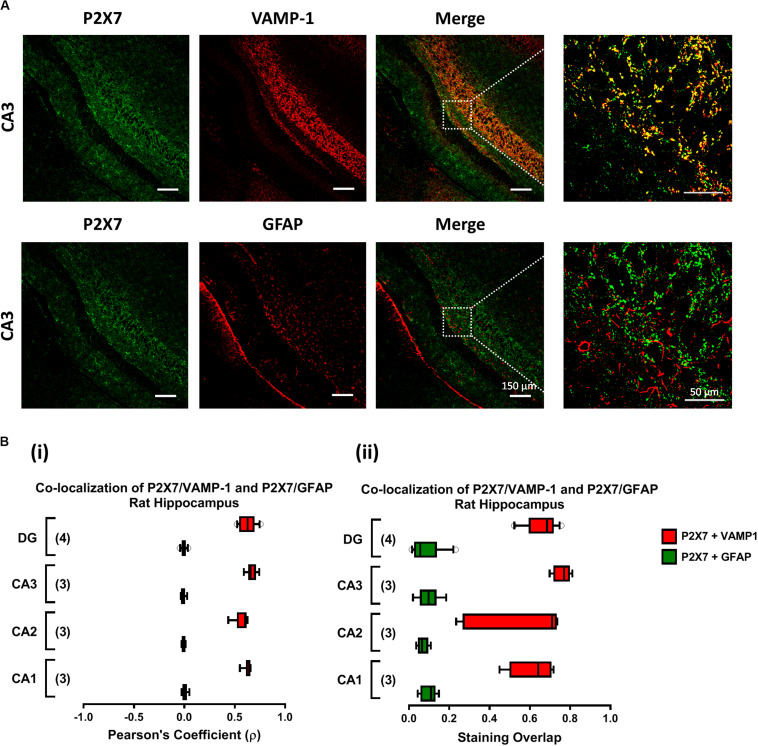
The P2X7 receptor is present predominantly on nerve terminals of the rat hippocampus. Panel **(A)** shows the immunolocalization of the P2X7 receptor in confocal micrographs of the CA3 region of rat hippocampal slices (scale bar = 150 μm); right hand-side panels show higher magnification images zoomed from the indicated regions (scale bar = 50 μm). Synaptic nerve terminals are stained with the vesicle-associated membrane protein 1 (VAMP-1 or synaptobrevin 1) and astrocytes are labeled with the glial fibrillary acidic protein (GFAP). Data show that the P2X7 receptor (green) localizes predominantly in VAMP-1-positive synaptic nerve terminals (upper panels), but not in cells positively staining with GFAP (lower panels); yellow staining denotes co-localization of P2X7 receptors (green) and type-specific cell markers (red). In panel **(B)**, fluorescence intensity scatter plots were used to estimate staining co-localization by calculating the Pearson’s Coefficient (ρ, **Bi**) and the staining overlap **(Bii)** scores; represented are “box-and-whiskers” graphs plotting data from three to four individuals performed in triplicate. These parameters were automatically calculated per image and were used to quantify the colocalization of the P2X7 receptor either with VAMP-1 or with GFAP (yellow staining) in different hippocampal subregions, namely CA1, CA2, CA3 and dentate gyrus (DG).

Previously, our group demonstrated that MP (300 μM) favors the release of ATP from resting motor nerve terminals (Oliveira et al., 2015) and the nucleotide acting via ionotropic P2X7 receptors can differentially affect GABA and Glu release (and uptake) from isolated nerve terminals of the rat cerebral cortex (Barros-Barbosa et al., 2015, 2018). Confocal micrographs depicted in Figure 5 show that the fairly specific antibody against the C-terminal of the rat P2X7 receptor (#APR-004) co-localizes extensively with the synaptic nerve terminal marker, VAMP-1, but no color merge was detected in GFAP-positive astrocytic cells of the rat hippocampus. The co-localization scores assessed by calculating the staining overlap and the Pearson’s coefficient (ρ) for each confocal micrograph are in agreement with our findings in the rat cerebral cortex (Barros-Barbosa et al., 2015), thus confirming that the P2X7 receptor is present on nerve terminals of the various sub-regions of the rat hippocampus, namely CA1, CA2, CA3 and dentate gyrus (Figure 5). This prompted us to test the effect of MP (300 μM) on [^3^H]GABA and [^14^C]Glu release measured synchronously from isolated nerve terminals of the rat hippocampus in the presence of a selective P2X7 receptor antagonist, A-438079.

**FIGURE 6:**
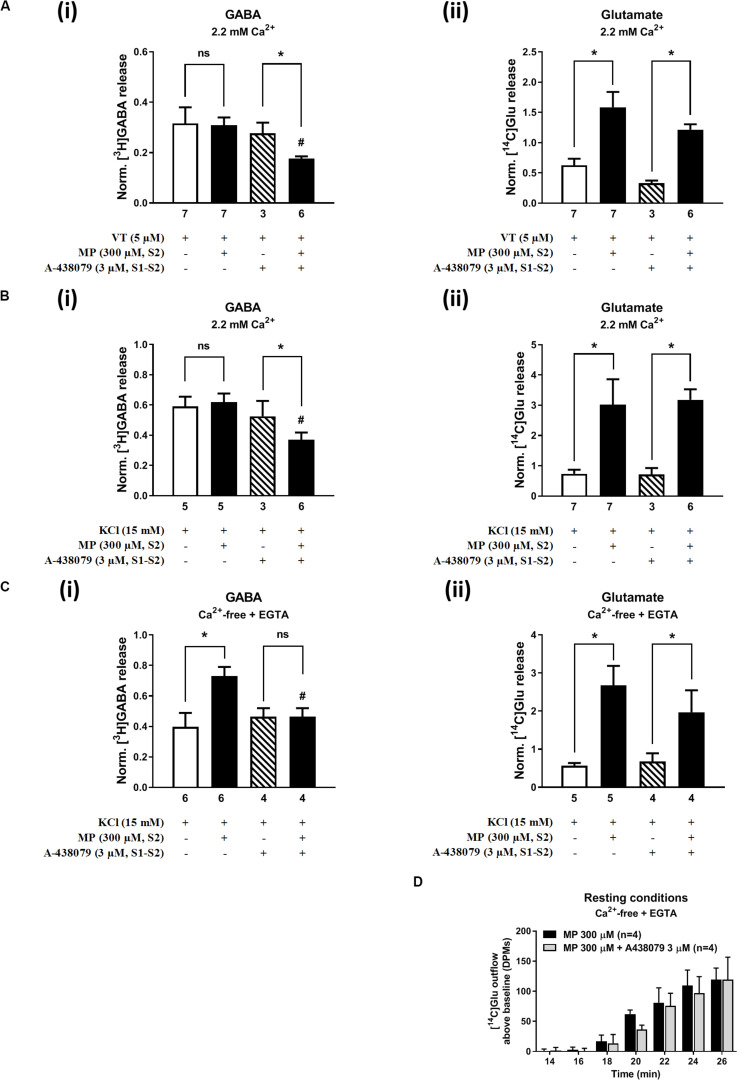
Blockage of the ATP-sensitive P2X7 receptor decreases depolarization-evoked [^3^H]GABA release from hippocampal nerve terminals in the presence of methylprednisolone (MP), without affecting the release of [^14^C]Glu. In panels **(A–C)**, ordinates represent the normalized [^3^H]GABA **(i)** and [^14^C]Glu **(ii)** release (S2/S1 ratio) from hippocampal nerve terminals depolarized with VT (5 μM, **A**) and high KCl (15 mM) applied in normal Ca^2+^ (2.2 mM, **B**) conditions and after removing Ca^2+^ (plus EGTA, 1 mM, **C**) from the incubation medium. MP (300 μM) was applied to the superfusion fluid 15 min before S2; the P2X7 receptor antagonist, A-438079 (3 μM), was present throughout the assay, including S1 and S2. Panel **(D)** illustrates the outflow of [^14^C]Glu above baseline over time caused by MP (300 μM) in the absence and in the presence of A-438079 (3 μM) in Ca^2+^-free (plus EGTA, 1 mM) conditions. The results are expressed as mean ± SEM; the n number of individual experiments per condition is shown below each bar. In panels **(A–C)**, *P < 0.05 and ^#^*P* < 0.05 (one-way ANOVA, uncorrected Fisher’s LSD with single pooled variance) represent significant differences compared to control conditions (i.e., drugs in S1 and S2) and to the effect of MP alone, respectively; ns, non-significant; the computed *F* ratios were 2.35 **(Ai)**, 3.35 **(Aii)**, 2.27 **(Bi)**, 12.39 **(Bii)**, 4.26 **(Ci)**, and 8.94 **(Cii)**. In panel **(D)**, *P < 0.05 (unpaired Student’s *t*-test, corrected for multiple comparisons using the Holm–Sidak method) represent significant differences as compared to the effect of MP alone.

On its own, A-438079 (3 μM) was unable to modify (*P* > 0.05) the release of [^3^H]GABA and [^14^C]Glu from isolated nerve terminals depolarized with VT (5 μM) (Figure 6A) and high KCl (15 mM) (Figure 6B); the S2/S1 ratio of KCl-induced [^3^H]GABA and [^14^C]Glu release when A-438079 (3 μM) was applied 15 min before S2 was 0.52 ± 0.05 (vs. a control of 0.59 ± 0.08, *n* = 6; *P* > 0.05) and 0.78 ± 0.15 (vs. a control of 0.72 ± 0.15, *n* = 6; *P* > 0.05), respectively. Blockage of the P2X7 receptor with A-438079 (3 μM) attenuated (*P* < 0.05) VT- and high KCl-induced [^3^H]GABA release from hippocampal nerve terminals challenged with MP (300 μM), without affecting the coincidental release of [^14^C]Glu (Figures 6A,B, respectively). More interestingly, pre-treatment with A-438079 (3 μM) abolished MP (300 μM)-induced facilitation of [^3^H]GABA release from KCl-depolarized nerve terminals in Ca^2+^-free conditions (Figure 6Ci), without affecting the facilitatory effect of the corticoid on [^14^C]Glu release, both under resting conditions (Figure 6D) and during depolarization of rat hippocampal nerve terminals with high KCl (15 mM) (Figure 6Cii).

The P2X7 receptor agonist, 2′(3′)-*O*-(4-benzoylbenzoyl)ATP (BzATP, 300 μM), increased from 0.86 ± 0.07 to 1.16 ± 0.08 (*n* = 6, *P* < 0.05) the release of [^3^H]GABA from hippocampal nerve terminals depolarized with high KCl in normal external Ca^2+^ conditions, but this effect was not observed regarding the release of [^14^C]Glu (data not shown). The facilitatory effect of BzATP (300 μM) on KCl-induced [^3^H]GABA release was prevented by A-438079 (3 μM, 0.78 ± 0.08, *n* = 6), thus confirming the involvement of ATP-sensitive P2X7 receptors.

**FIGURE 7:**
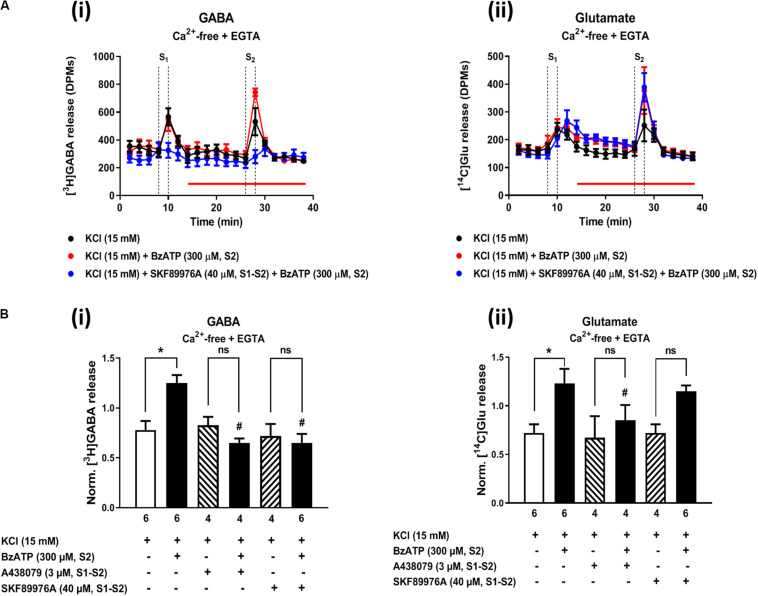
Activation of the P2X7 receptor with BzATP increases KCl-evoked [^3^H]GABA and [^14^C]Glu release from hippocampal nerve terminals in Ca^2+^-free conditions. Panel **(A)** shows [^3^H]GABA **(Ai)** and [^14^C]Glu **(Aii)** outflow from rat hippocampal nerve terminals over time in Ca^2+^-free (plus EGTA, 1 mM) conditions; nerve terminals were depolarized twice (S1 and S2) with high KCl (15 mM) applied for 2 min (dotted vertical lines). BzATP (300 μM) was applied to the superfusion fluid 15 min before S2 (red horizontal line) either in the absence or in the presence of the GAT-1 transport inhibitor, SKF 89976A (40 μM), or the P2X7 receptor antagonist, A-438079 (3 μM), which were present throughout the assay, including S1 and S2. In panel **(B)**, ordinates represent the normalized [^3^H]GABA **(Bi)** and [^14^C]Glu **(Bii)** release (S2/S1 ratio) from depolarized hippocampal nerve terminals in Ca^2+^-free conditions (plus EGTA, 1 mM). The results are expressed as mean ± SEM; the *n*, number of individual experiments per condition is shown below each bar. *P < 0.05 and ^#^*P* < 0.05 (one-way ANOVA, uncorrected Fisher’s LSD with single pooled variance) represent significant differences compared to control conditions (i.e., drugs in S1 and S2) and to the effect of BzATP alone, respectively; ns, non-significant; the computed *F* ratios were 7.19 **(Bi)** and 3.94 **(Bii)**.

In a previous study, we demonstrated (1) that the facilitatory action of the ionotropic P2X7 receptor is enhanced after removal of Ca^2+^ from incubation media (like that observed here with MP; see Figures 2Ci, 4Av), and (2) that under Ca^2+^-free conditions the release of [^3^H]GABA results mainly from the collapse of the transmembrane Na^+^ gradient and, subsequent, reversal of the GAT-1-mediated transport (Barros-Barbosa et al., 2018). Figure 7 shows that inhibition of GAT-1 with SKF 89976A (40 μM) significantly (*P* < 0.05) attenuated BzATP (300 μM)-induced facilitation of [^3^H]GABA release from KCl-depolarized hippocampal nerve terminals in Ca^2+^-free conditions (Figures 7Ai,Bi), without affecting facilitation of [^14^C]Glu release by the nucleotide (Figures 7Aii,Bii). SKF 89976A (40 μM) mimicked the inhibitory effect of the P2X7 receptor antagonist, A-438079 (3 μM), on MP (300 μM)-induced facilitation of [^3^H]GABA release under similar experimental conditions (Figures 7Ai,Bi).

## Discussion

Data show here for the first time that MP favors the release of [^14^C]Glu both from resting as well as from depolarized nerve terminals of the adult rat hippocampus, with no effect on the coincidental release of [^3^H]GABA under physiological conditions. The mechanism underlying facilitation of [^14^C]Glu release by MP has several important features. First, it involves the putative activation of membrane-bound corticosteroid receptors sensitive to mifepristone and spironolactone. Second, MP causes a progressive increase in [^14^C]Glu outflow starting immediately (within a few minutes) after application of the corticosteroid, which is compatible with a fast non-genomic effect. Third, facilitation of evoked [^14^C]Glu release is relatively independent of the depolarizing agent, either high KCl or VT. Fourth, MP-induced [^14^C]Glu release is abrogated by dissipation of the Na^+^ gradient across the plasma membrane, but is little affected by changes in extracellular Ca^2+^, suggesting that it results from non-vesicular release operated by Na^+^-sensitive high-affinity EAAT transporters working in the reverse mode. The molecular mechanism responsible for this new working hypothesis has no parallel in the literature and certainly deserve to be explored in future studies. Regarding depolarization-evoked [^3^H]GABA release in the presence of MP, data indicate that it may involve a yet unraveled (direct or indirect) interplay between low affinity GR and P2X7 receptors activation taking into consideration the inhibitory effects obtained with mifepristone and A-438079, respectively, and the fact that it is relatively sensitive to removal of Ca^2+^, but not Na^+^, from the incubation medium.

Corticosteroid alterations of neuronal excitability throughout the brain have been mostly attributed to fast non-genomic effects (Di et al., 2003, 2005, 2009; Karst et al., 2005; Groc et al., 2008; Groeneweg et al., 2012). This theory is supported by our data considering (1) the (min) timescale required to observe changes in [^14^C]Glu release and (2) the fact that we have used synaptophysin-enriched isolated nerve terminals remaining after rejecting the nucleated neuronal cell bodies and GFAP-positive glial cells of the rat hippocampus, which otherwise can confound data interpretation in whole cell preparations. The involvement of plasma membrane-bound GR and MR is highly likely because these receptors can be blocked by mifepristone and spironolactone, respectively, and both receptors were previously identified by electron microscopy in synaptosomal extracts and neuronal membranes of several brain regions (Johnson et al., 2005; Groeneweg et al., 2012), including the rat hippocampus (Wang and Wang, 2009). In keeping with our assumption, others have found that the facilitatory effect of corticosterone (and its cell impermeable conjugate with bovine serum albumin) on depolarization-induced [^14^C]Glu release in the hippocampus was also blocked by mifepristone (Karst et al., 2005; Wang and Wang, 2009), as well as by spironolactone (Groc et al., 2008). Notwithstanding these facts, the presence of functional GR receptors in the plasma membrane have been questioned mostly because these receptors have not yet been cloned and their downstream signaling pathways are still unknown.

In our hands, MP-induced increase in basal [^14^C]Glu release was mimicked by the five-fold more potent GR agonist, dexamethasone, and it was more sensitive to blockage with mifepristone than with spironolactone, suggesting a predominant role of low affinity GRs on adult rat hippocampal nerve terminals acting to facilitate glutamate release (see also e.g., Wang and Wang, 2009). Prevention of MP-induced facilitation of [^14^C]Glu release from depolarized nerve terminals by both corticosteroid antagonists raises questions about the selectivity of spironolactone (500 nM) for high affinity MR and whether it can also perturb the action of coexisting low affinity GR that more likely facilitate hippocampal glutamate transmission (Groc et al., 2008). The way GRs activation might cause reversal of DL-TBOA-sensitive Na^+^-dependent EAAT transporters in order to release [^14^C]Glu from pre-synaptic nerve terminals is still unknown, but it certainly deserves attention in future studies.

To the best of our knowledge, this is the first study reporting effects of corticosteroids, namely MP, on [^3^H]GABA release from hippocampal nerve terminals measured in parallel and under the same experimental conditions as those used to quantify the release of [^14^C]Glu. However, in contrast to the more straightforward excitatory effect of MP on [^14^C]Glu outflow, MP-induced facilitation of [^3^H]GABA release was only evident when rat hippocampal nerve terminals were depolarized with high KCl (but not VT) in external Ca^2+^-free conditions (see Figures 2C, 4Av), a situation that is very similar to that verified after paroxysmal neuronal activity (e.g., epileptic crises) where the extracellular concentration of Ca^2+^ falls by more than 90% (Engelborghs K. et al., 2000; Engelborghs S. et al., 2000). Although under normal Ca^2+^ conditions MP failed to affect GABA outflow, unexpectedly the amount of [^3^H]GABA released during depolarization of hippocampal nerve terminals in the presence of MP was significantly reduced upon blocking either MRs with spironolactone or ATP-sensitive ionotropic P2X7 receptors with A-438079. We have so far no reasonable explanation to these experimental findings, but somehow blockage of MR and P2X7 receptors may unbalance depolarization-induced [^3^H]GABA release in the presence of the corticoid decreasing the transmitter release probability when external Ca^2+^ is available, but not in stressful (low Ca^2+^) conditions. One may hypothesize that MP might favor opening of the ATP-sensitive P2X7 receptor pore by a yet unknown mechanism leading to Na^+^ influx into hippocampal nerve terminals and, subsequent, disruption of the transmembrane Na^+^ gradient differentially affecting GABA and Glu release, as previously demonstrated (Barros-Barbosa et al., 2018). The preferring effect of the P2X7 receptor on [^3^H]GABA release was confirmed using the ATP analog, BzATP, and might involve reversal of the neuronal GAT1 transporter sensitive to SKF 89976A (see e.g., Sperlágh et al., 2002), a situation that is much more likely to occur in the absence of external Ca^2+^ (Barros-Barbosa et al., 2018). The higher sensitivity for GABA than for Glu release under low Ca^2+^ conditions is probably because the GAT1 reversal potential is close to the resting potential of the neuronal membrane (Zerangue and Kavanaugh, 1996; Richerson and Wu, 2003; Allen et al., 2004; Wu et al., 2006).

Although it is generally assumed that low extracellular Ca^2+^ reduces vesicular exocytosis of neurotransmitters, an inverse correlation between Ca^2+^ concentration in the extracellular fluid and depolarization-evoked amino-acid transmitters release may be observed (see e.g., Levi et al., 1980; Cunningham and Neal, 1981; Minc-Golomb et al., 1988; Santos and Rodriguez, 1992; Rassner et al., 2016; Barros-Barbosa et al., 2018). While [^3^H]GABA release may be operated by a Ca^2+^-dependent pathway when extracellular Ca^2+^ is available, this mechanism may shift toward the reversal of the GAT1 transporter in low Ca^2+^ conditions (Barros-Barbosa et al., 2018; see also Figure 7) and, thus, became potentiated by GRs activation with MP. A different scenario is verified regarding the release of [^14^C]Glu, since the facilitatory effect of MP was consistently verified both in the absence and in the presence of external Ca^2+^, except when hippocampal nerve terminals were depolarized by the Na^+^ channel activator, VT, which keeps the channel pore persistently opened.

This new vision regarding the molecular mechanisms underlying the differential role of corticosteroids, namely MP, on amino-acid transmitters release, together with the fact that hippocampal neurons abundantly express both MRs and GRs (Karst et al., 2005; Joëls et al., 2012), may impact on the rationale for designing new strategies for better treatment of pathological conditions affecting the hippocampus, specifically drug-refractory epilepsy, post-traumatic stress, memory deficits and neurodegenerative diseases. In a recent study from our group, it was proposed that low doses of MP given for a short period of time favored *in vivo* aversive memory persistence and this was correlated with significant gains in *in vitro* hippocampal LTP (de Vargas et al., 2017). Hippocampal LTP is highly dependent on neuronal plasticity phenomena that are commonly associated with glutamatergic neurotransmission strengthening. We demonstrate here that MP significantly increases the neuronal release of [^14^C]Glu, both during resting and upon depolarization of hippocampal nerve terminals, without significantly affecting the release of the inhibitory neurotransmitter. Fast (non-genomic) onset enhancement of glutamatergic neurotransmission has also been observed in the CA1 hippocampal area (Karst et al., 2005). Strengthening of glutamatergic synaptic potentiation by reversibly increasing AMPA receptor-mediated miniature excitatory postsynaptic current (mEPSC) frequency was also verified by other authors upon application of corticosterone (and its membrane-impermeable conjugate with bovine serum albumin) to CA1 pyramidal neurons (Groc et al., 2008; see also Karst et al., 2005). Likewise, low nanomolar concentrations of corticosterone rapidly increased GluR2-AMPAR surface diffusion at the post-synaptic region also favoring glutamatergic synaptic potentiation.

Data from the present study clearly indicate that MP increased the release of [^14^C]Glu both under resting conditions and during depolarization of hippocampal nerve terminals from adult rats. The closest mention we could found about spontaneous glutamate release induced by steroid hormones was in few reports showing that pregnenolone sulfate (PREGS; a neuroactive steroid hormone) dose-dependently increased the frequency of mEPSCs in immature neurons of the hippocampus (Meyer et al., 2002; Chen and Sokabe, 2005; Mameli et al., 2005), yet no direct assessment to glutamate release has been reported in the hippocampus of adult animals. Evidence that resting [^14^C]Glu release induced by MP is Na^+^-dependent, yet Ca^2+^-independent, is in favor of the participation of DL-TBOA-sensitive Na^+^-dependent high-affinity EAAT membrane transporters operating in the reverse mode, which is a novelty provided by our study.

Our group demonstrated that at the cholinergic neuromuscular synapse MP facilitates acetylcholine release indirectly by increasing ATP release and, subsequent, adenosine formation resulting in increased activation of facilitatory A_2*A*_ receptors (Oliveira et al., 2015). On its own, steroids may induce rapid (non-genomic) gating of several ionotropic receptors, including ATP-sensitive P2X receptors (Codocedo et al., 2009). Interestingly, synthetic testosterone derivatives have been shown to positively modulate the activity of P2X2 and P2X4, but not P2X7, receptors in heterologous systems (see e.g., Sivcev et al., 2019). It remains, however, to be elucidated whether (1) MP can allosteric modulate ATP-induced P2X7 receptor gating, and if (2) the putative allosteric binding site for MP at the P2X7 receptor pore is specifically inhibited by GR antagonists. We are also aware that steroids may activate plasma membrane receptors coupled to G proteins (Mizota et al., 2005; Schiess and Partridge, 2005), which may directly or indirectly modify ATP-gated P2X receptor currents via downstream intracellular signaling pathways. Notwithstanding the fact that we did not prove that MP induced the release of ATP, which by acting via low affinity ionotropic P2X7 receptors may favor transmitters outflow from rat hippocampal nerve terminals, the P2X7 receptor antagonist, A-438079, significantly attenuated depolarization-evoked [^3^H]GABA, but not [^14^C]Glu, release in the presence of MP. This result is in keeping with previous findings in nerve terminals of the rat cerebral cortex showing that the P2X7 receptor activation unbalances GABAergic vs. glutamatergic neurotransmission by differentially affecting high affinity [^3^H]GABA and [^14^C]Glu uptake (Barros-Barbosa et al., 2015) and release (Barros-Barbosa et al., 2018) in an extracellular Ca^2+^-sensitive manner. Under the present experimental conditions, isolated nerve terminals of the rat hippocampus are the only possible source of endogenous ATP required to activate P2X7 receptors in the presence of MP.

Despite activation of hippocampal P2X7 receptor has been implicated in physiological processes, such as learning and memory, it may gain a different meaning under pathological conditions, such as meso-temporal lobe epilepsy, where the expression of this receptor is upregulated (Jimenez-Pacheco et al., 2013; Barros-Barbosa et al., 2016). Thus, strengthening the inhibitory GABAergic neurotransmission, without significantly affecting Glu release, by P2X7 receptor activation triggered by corticosteroid receptors may become apparent during paroxysmal neuronal firing where extracellular Ca^2+^ is low (Engelborghs K. et al., 2000; Engelborghs S. et al., 2000). We foresee that these previously unpredicted differential roles of corticosteroids on GABA and glutamate release may change our therapeutic approach to improve cognition and to avoid detrimental neuroexcitotoxicity in drug-refractory epilepsy and other neurological conditions. Thus, facilitation of Glu release strengthen previous assumptions that MP may act as a cognitive enhancer, while crosstalk with overexpressed ATP-sensitive P2X7 receptors may provide therapeutic benefits by increasing the GABAergic inhibitory drive during paroxysmal epileptic crisis and/or neuroinflammatory insults of the adult brain.

## Data Availability Statement

All datasets generated for this study are included in the article/supplementary material.

## Ethics Statement

The animal study was reviewed and approved by the Ethics Committee and the Animal Welfare Responsible Organism of ICBAS-UP (Decision no 224/2017).

## Author Contributions

PC-d-S supervised the project. RN, ML, and PC-d-S designed the experiments. RN and PC-d-S wrote the manuscript. RN, AC-R, FF, and ML carried out the experimental work. RN, AC-R, FF, ML, and PC-d-S interpreted data and commented on the manuscript at all stages. All authors contributed to the article and approved the submitted version.

## Conflict of Interest

The authors declare that the research was conducted in the absence of any commercial or financial relationships that could be construed as a potential conflict of interest.
